# Different Classes of Phytohormones Act Synergistically to Enhance the Growth, Lipid and DHA Biosynthetic Capacity of *Aurantiochytrium* sp. SW1

**DOI:** 10.3390/biom10050755

**Published:** 2020-05-13

**Authors:** Yusuf Nazir, Hafiy Halim, Pranesha Prabhakaran, Xiaojie Ren, Tahira Naz, Hassan Mohamed, Shaista Nosheen, Kiren Mustafa, Wu Yang, Aidil Abdul Hamid, Yuanda Song

**Affiliations:** 1 Colin Ratledge Center for Microbial Lipids, School of Agriculture Engineering and Food Science, Shandong University of Technology, Zibo 255049, China; yusufnazir91@yahoo.com (Y.N.); hafiyhalim@gmail.com (H.H.); sha_6p@yahoo.com (P.P.); renxiaojie2020@163.com (X.R.); nazkhan658@gmail.com (T.N.); Hassanmohamed85@azhar.edu.eg (H.M.); shaista_nosheen@yahoo.com (S.N.); mustafakiran92@gmail.com (K.M.); neverhangsome@hotmail.com (W.Y.); 2 Department of Botany and Microbiology, Faculty of Science, Al-Azhar University, Assiut 71524, Egypt; 3 School of Biosciences and Biotechnology, Faculty of Science and Technology, Universiti Kebangsaan Malaysia, Bangi 43600, Malaysia

**Keywords:** phytohormones, growth, lipid, docosahexaenoic acid, reactive oxygen species, malondialdehyde, lipogenic enzymes, superoxide dismutase, catalase, *Aurantiochytrium* spp.

## Abstract

In the present study, the impact of eight phytohormones from six different classes on the growth, lipid and docosahexaenoic acid (DHA) biosynthetic capacity of *Aurantiochytrium* sp. SW1 (SW1) was evaluated. Kinetin (KIN), jasmonic acid (JA) and gibberellic acid (GA) significantly enhanced the growth and DHA production of SW1 by 16%–28% and 66%–84% in comparison to the control, respectively. The synergistic effect of these three phytohormones, evaluated by the response surface methodology (RSM), showed that a combination of 3.6 mg/L GA, 2.0 mg/L KIN and 20.0 mg/L JA further increased the growth and DHA production of SW1 by 16% to 28% and 22% to 36%, respectively, in comparison to the individual supplementation. The synergistic effect of these phytohormones was also shown to be time-dependent, where feeding at 24 h of cultivation led to 15%, 26% and 35% further increments in the biomass, lipid and DHA production in comparison to that of 0 h, respectively. The determination of stress markers, antioxidant enzymes and key enzymes involved in fatty acid biosynthesis aided to elucidate the potential mechanism underlying the improvement of growth and DHA production by SW1 at various times of feeding. Supplementation with the phytohormones at 24 h exhibited the maximum impact on reducing the level of reactive oxygen species (ROS) and malondialdehyde (MDA), as well as augmented the antioxidants (superoxide dismutase and catalase) and key metabolic enzymes involved in lipogenesis (malic, glucose-6-phosphate dehydrogenase and ATP-citrate lyase) in comparison to the control and other time points. This study signifies the potential application of phytohormones for improving the growth, lipid and DHA production in *Aurantiochytrium* spp.

## 1. Introduction

The dietary importance of long-chain polyunsaturated fatty acids (LCPUFAs), such as omega 3 and omega 6, in upholding human well-being and longevity has been proposed decades ago. These fatty acids are regarded as essential fatty acids due to the fact that the human body lacks certain enzymes to synthesize them. Thus, they must be obtained from the diet. Docosahexaenoic acid (DHA), one of the members of the omega-3 LCPUFAs, has attracted significant interest worldwide due to its vital role in human health and well-being. This fatty acid can naturally be found in mother’s milk owing to its importance for the development of the brain and eyes of an infant [1,2]. Furthermore, DHA is also proven for its effectiveness in curing several diseases, such as cancer, coronary heart disease, hypertension, depression, type 2 diabetes mellitus, atherosclerosis, thrombosis and many more [3,4]. Thus, DHA is widely used as a nutraceutical component in the food and feed market [4]. Currently, the commercial source of DHA is fish oil derived from cold-water fatty fish, such as tuna and salmon. However, several drawbacks have been found for fish oils, such as the presence of high saturated fatty acids, contamination by hazardous substances, fishy odor, low quantity DHA and complications in the purification process [5]. Therefore, an alternative DHA source to fish oils was explored.

Microbial oil, being produced in a controlled environment, is one of the current topics of massive research because it has many advantages compared to fish oil [6,7]. Marine heterotrophic protists, largely known as marine microalgae, especially *Aurantiochytrium* spp., have been shown to be excellent DHA producers. Members of the genus *Aurantiochytrium* are able to produce a substantial amount of lipid where DHA accounts for as much as 35%–55% of the total fatty acids (TFAs) [7,8]. Furthermore, DHA from *Aurantiochytrium* has also been proven to be safe for human consumption as it is free from the common algal toxins, such as domoic acid and prymnesin, produced by some members of its kingdom, such as Chromista [9]. Therefore, its applications as a novel food supplement and in infant formula have been recommended in many countries throughout the world. Nevertheless, major efforts and strategies need to be further explored to enhance the DHA production by this microorganism.

Improving the fermentation process as well as the stress-based strategies are the common approach to further enhance the lipid production in most microalgae, including *Aurantiochytrium*. Nitrogen starvation has been proven to be an effective stress-based technique to enhance the lipid content and productivity in microalgae. For example, Jia et al. [10] reported that under nitrogen depletion conditions, the lipid content in *Nannochloropsis oceanica* was about twofold higher than that in normal medium. In addition, Ma et al. [11] reported a significant increment in DHA content in total fatty acids when *Aurantiochytrium* sp. BLH was induced with cold stress. Nevertheless, these stress-based techniques often resulted in compromised growth and elevated formation of ROS, which led to the loss of protein function and even cell death [12,13]. The addition of antioxidants, such as ascorbic acid and sesamol, as well as the overexpression of superoxide dismutase (SOD) in *Schizochytrium* spp. have successfully alleviated the oxidative stress and increased the DHA content in the microalgae [13,14,15]. However, the primary effect of the antioxidants was only to reduce the oxidative damage, rather than inducing the growth, lipid and DHA biosynthesis [16].

Phytohormones were recently reported to pose stimulating effects on the growth and biosynthesis of fatty acids in microalgae [17,18]. Phytohormones have also been proposed not only to reduce the oxidative damage in cells but also to increase the growth and lipid production by controlling the internal biochemical pathways [19]. For example, the addition of 20 mg/L fulvic acid (FA) and 1 mg/L ethylenediaminetetraacetic acid (EDTA) resulted in a 36.4% and 34.4% increase in biomass and lipid yield in *Schizochytrium* spp., which exhibited a lower ROS and MDA level accompanied with higher superoxide dismutase (SOD) and catalase (CAT) activity in comparison to the control [16]. Furthermore, Yu et al. [17] revealed that the addition of auxins indole acetic acid (IAA) and naphthylacetic acid (NAA) increased the lipid productivity up to threefold under nitrogen-depleted conditions as compared to standard BG-11 medium, by modulating the oxidative damage caused by ROS during the cultivation. Moreover, the addition of 4 mg/L GA has been shown to significantly enhance the production of DHA in *Aurantiochytrium* spp. [20]. Nevertheless, most of the studies were just focusing on evaluating the individual effect of phytohormone supplementation while the synergistic effect of phytohormones from different classes, their interaction with the oxidative stress as well as the key metabolic enzymes are still sporadic, particularly in *Aurantiochytrium* spp.

Therefore, in this study, the synergistic effect of different classes of phytohormones on the cellular growth, lipid content as well as DHA biosynthetic capacity of *Aurantiochytrium* sp. SW1 was investigated. Furthermore, the activities of the antioxidant defense systems, the key enzymes involved in lipid biosynthesis as well as the levels of the oxidative damage indicators, including ROS and MDA, were determined to explore the mechanisms guiding the physiological and molecular changes triggered at different time points of phytohormone supplementation.

## 2. Materials and Methods

### 2.1. Organism and Culture Conditions

The microorganism used in this study is *Aurantiochytrium* sp. SW1 (GenBank: KF500513), provided by the Microbial Physiology Lab, School of Biosciences and Biotechnology, Universiti Kebangsaan Malaysia, as well as the Colin Ratledge Centre for Microbial Lipid, Shandong University of Technology, and has been deposited in UNiCC UPM under the accession number UPMC 963. This organism was maintained on seawater nutrient agar (SNA) as slant culture, which contained 28 g/L nutrient agar and 17.5 g/L artificial seawater accounting for 50% (*w*/*w*) salinity. Seed cultures were prepared by inoculating 100 mL of a seeding broth with a strip of SNA slant agar containing approximately ten colonies of 48-h SW1 cells in 500 mL Erlenmeyer flasks. Seed cultures were then incubated at 28 °C for 48 h with an agitation rate of 200 rpm. The medium used in the seed cultures contained 60 g/L glucose, 2 g/L yeast extract, 8 g/L monosodium glutamate (MSG) and 6 g/L artificial sea salt. The composition of the sea salt used in this study was described by Manikan et al. [21]. Seed culture with an inoculum size of 10% (*v*/*v*) was inoculated into the production medium as described in Section 2.2, Section 2.3 and Section 2.4. The cultures were then incubated at 28 °C for 96 h and 144 h.

### 2.2. Screening on the Impact of Phytohormones Supplementation on the Growth and DHA Production of SW1

Eight analytically pure phytohormones from six different classes were used in the screening study, as shown in Table 1. The stock solution of all the phytohormones was initially prepared by dissolving a known concentration of each phytohormone with dimethyl sulfoxide (DMSO) and the reagents were stored at 4 °C until use. The stock solutions were then diluted accordingly with the concentration shown in Table 1 in DMSO and sterilized using 0.22 μm polyvinylidene fluoride or a polyethersulfone syringe filter (Millipore, MA, USA) before adding into the cultivation media. The screening experiment was carried out in a 250 mL flask with 50 mL of working volume. The same amount of DMSO was added in the control experiments. The cultures were then cultivated for 96 h with a 200 rpm agitation speed at 28 °C. The biomass and DHA were then assayed. Data were analyzed using the mean and standard deviation of the triplicate experiments.

### 2.3. Synergistic Effect and Optimization of KIN, JA and GA Concentration Using the Response Surface Methodology (RSM)

An RSM based on the central composite design (CCD-response surface; Design Expert Software (DOE), version 11.0, Stat-Ease, USA) was employed to evaluate the synergistic effect of KIN, JA and GA supplementation on growth and DHA production of SW1. The chosen ranges in concentration were set based on the optimal ranges obtained in the screening experiment, represented as follows: KIN, 1–3 mg/L; JA, 10–30 mg/L; and GA, 2–5 mg/L. In addition, statistical analysis using ANOVA with the DOE software was used to estimate the optimal concentration of the KIN, JA and GA for maximum growth and DHA production by SW1. The coefficients in the second-order polynomial (Equation (1)) were calculated by multiple regression analysis of the experimentally obtained results.

(1) $$Y=b_{0}^{\prime }+\overset{n}{\underset{i=1}{\sum }}b_{i}X_{i}+\overset{n}{\underset{i=1}{\sum }}b_{ii}X_{i}^{2}+\overset{n}{\underset{i=1}{\sum }}\cdot \overset{n}{\underset{j\geq 1}{\sum }}b_{ij\;}X_{i}X_{j}\;\;\;\;\;\;\;\;$$

where *Y* is the predicted response; *b’_0_* is the constant coefficient; *bi* is the linear coefficient; *b_ij_* is the interaction coefficient; *b_ii_* is the quadratic coefficient; and *X_i_* and *X_j_* are coded values. Additionally, three-dimensional (3D) response surface and contour plots were constructed for visual observation of the trend of the maximum responses and the interaction effects of the significant variables on the responses.

### 2.4. Supplementation of the Optimal Concentration of KIN, JA and GA at Different Time Points

The optimal concentration of KIN, JA and GA obtained from the RSM experiment was supplemented at 0, 12, 24 and 48 h of cultivation and the impact on the growth, lipid and overall DHA production was assayed. The cultures were then cultivated for 144 h with a similar condition, as shown in Section 2.2. The data were analyzed using the mean and standard deviation of triplicate experiments.

### 2.5. Cell-Free Extracts Preparations and Enzyme Assays

Harvested microalgal cells were suspended in an extraction buffer [22] and subsequently disrupted by ultrasonication (Scientz-II D sonifier) at 400 W for 5 s intervals with cooling in between on ice for 10 min. The cells were then centrifuged at 12,000× *g* for 10 min at 4 °C using an Eppendorf centrifuge 5810 R and the supernatant was filtered with Whatman No. 1 filter paper to recover the cell-free extract. The supernatant containing cytoplasmic and mitochondrial enzymes was subjected to enzyme activity analysis. The activities of four enzymes, namely malic enzyme (ME), ATP: citrate lyase (ACL), glucose-6-phosphate dehydrogenase (G-6-PDH) and NADP^+^-isocitrate dehydrogenase (ICDH), were determined using continuous assays following the oxidation and reduction of NAD(P)(H) at 340 nm and 30 °C. The change in absorbance was followed continuously for 10 min using software (UVPROBE2.31). Specific activity is expressed as nmol/min·mg protein. SOD and catalase (CAT) activities were determined using an assay kit (Beyotime Institute of Biotechnology, Shanghai, China) according to the manufacturer’s instructions. The specific activity was defined as unit/mg protein. One unit of enzyme activity (U) equal to “the amount of enzyme, required to produce 1 mol enzymatic reaction product in 1 min”. The protein concentration was determined using the Bradford method with BSA as a standard [23].

### 2.6. Reactive Oxygen Species (ROS) Determination

The intracellular ROS levels were determined by using the Reactive Oxygen Species Assay Kit (Beyotime Institute of Biotechnology, Shanghai, China) according to the manufacturer’s instructions. Briefly, 1 mL of the SW1 culture was harvested and washed with PBS buffer. A diluted dichloro-dihydro-fluorescein diacetate (DCFH-DA) probe was then added into the cell suspension and incubated at 37 °C for 30 min. The excess probe was washed twice with PBS buffer to ensure only the intracellular fluorescence was measured. Fluorescence intensity was detected by a fluorescence spectrophotometer with the excitation and emission wavelengths at 485 and 535 nm, respectively.

### 2.7. Lipid Peroxidation Assay (MDA Assay)

The lipid peroxidation level was determined by measuring the malondialdehyde (MDA) equivalent according to the method proposed by Heath and Packer [24]. Briefly, 1 mL of the harvested cells were disrupted, homogenized in 5% (w/v) trichloroacetic acid (TCA) and were then centrifuged at 10,000× *g* for 10 min at 4 °C. The supernatant was then mixed with 0.67% (w/v) thiobarbituric acid (TBA) solution and boiled for 20 min. The absorbance of the reaction mixture was recorded at 532 and 600 nm. The absorbance value recorded at 532 nm was subtracted by the non-specific absorbance value recorded at 600 nm, and the MDA equivalent content was measured using the 155 mM/cm extinction coefficient.

### 2.8. Determination of Dry Cell Weight (DCW)

Algal cells were harvested by centrifugation at 10,000× *g* for 10 min using an Eppendorf centrifuge 5810 R. The cells were then washed twice with sterile distilled water. Cell samples were then freeze-dried for 48 h and weighed. Biomass obtained was expressed as gram dried cell per liter of cultivation medium.

### 2.9. Lipid Extraction and Fatty Acid Analysis

Lipid extraction was performed using chloroform–methanol (2:1, *v*/*v*), as described by Folch et al. [25]. The extract was vaporized at room temperature and dried in a vacuum desiccator until a constant weight was attained. Fatty acid compositions of the samples were determined as fatty acid methyl esters (FAMEs) by gas chromatography (HP 5890) equipped with a capillary column (BPX 70, 30 cm, 0.32 μm). FAME was prepared by dissolving 0.05 g of the sample in 0.95 mL hexane, and the mixture was added to 0.05 mL of 1 M sodium methoxide. The injector was maintained at 200 °C. Then, 1 μL of the sample was injected using helium as a carrier gas with a flow rate of 40 cm^3^ min^−1^. The temperature of the GC column was gradually increased at 7 °C min^−1^ from 50 (5 min hold) to 200 °C (10 min hold). Fatty acid peaks were identified using Chrome Leon chromatography software (Dionex, Sunnyvale, California, USA). FAMEs were identified and quantified by comparison with the retention time and peak areas of fatty acid standards from SUPELCO (Bellefonte, PA, USA).

## 3. Results and Discussions

### 3.1. Screening of Phytohormones for Improved Growth and DHA Production of SW1

In order to identify and evaluate the potential effect of phytohormone supplementation on the growth and the overall DHA production of SW1, a screening experiment with eight phytohormones from six different classes, including auxin, cytokinin, gibberellin, signal transducer, melatonin and amine, was conducted (Table 1). The selection of phytohormones and their concentration ranges were based on our preliminary screening as well as the previous studies from miscellaneous organisms [16,17,18,19,20,26,27,28]. Each of the phytohormones was used in eight different concentrations, as shown in Table 1. Meanwhile, the strain cultured with the same amount of solvent (DMSO) was set as the control. Nevertheless, no significant effect on either biomass or DHA production was observed in SW1 when supplemented with ABA and BNAO, unlike what was reported in *Cryptheconidium cohnii* where both of these phytohormones played a significant role in enhancing its lipid and DHA production [28]. Therefore, ABA and BNAO were excluded from further analysis.

**Figure 1:**
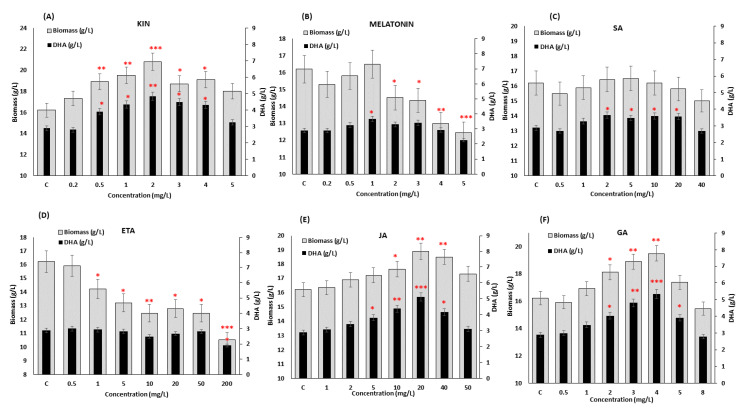
Screening of the effect of different concentrations of (**A**) KIN, (**B**) Melatonin, (**C**) SA, (**D**) ETA, (**E**) JA and (**F**) GA on biomass and DHA production in SW1 after 96 h of cultivation. Values and error bars represent the means and the standard deviations of triplicate experiments (*n* = 3). (*), (**) and (***) represents the statistical significance with respect to control values at *p* < 0.05, *p* < 0.01 and *p* < 0.001, respectively. The C represents the control of the experiment where no phytohormones were added to the medium.

The supplementation with phytohormones exhibited a significant impact on the growth and DHA production in SW1 (Figure 1). Nevertheless, only three phytohormones, namely GA (4 mg/L), KIN (2 mg/L) and JA (20 mg/L), posed a very significant positive impact (*p* < 0.01 and *p* < 0.001) on both biomass and DHA production, while melatonin and ETA, when exceeding a certain concentration, resulted in a significant negative impact on both responses. On the other hand, supplementation with SA only affected the DHA production, but not the growth of the SW1 (Figure 1). Supplementation with KIN at the concentration of 2 mg/L exhibited a maximum impact on the growth of the SW1, which was 27.90% ± 2.63% higher than that of the control group. On the other hand, supplementation of 4 mg/L GA and 20 mg/L JA increased the biomass production up to 20.16% ± 1.90% and 16.34% ± 1.54%, lower than what was observed in KIN. The positive impact of KIN supplementation in augmenting the growth of SW1 was also found to be similar to what was reported in *N. oceanica*, *Dunaliella salina* and *Heamatococus pluvialis* [26,29,30]. KIN is a group of natural cytokinins produced as growth regulators in plants and algae, and is an important inducer of the initiation of cell division. In addition, Lu et al. [26] reported that the exogenous addition of cytokinin stimulated cell cycle progression in *N. oceanica,* which resulted in an improved growth rate regardless of the nitrogen availability by modulating the regulatory protein of cytokinin receptor CR2. Similar mechanisms and responses may also be involved in SW1, explaining the higher biomass production obtained with KIN supplementation. Nevertheless, further research is required to confirm the claim.

Yet, the impact on the DHA production after KIN supplementation was not as substantial as what was observed in JA and GA where the addition of 20 mg/L JA and 4 mg/L GA resulted in 5.10 ± 0.44 and 5.34 ± 0.43 g/L DHA, respectively, which were approximately 6%–10% higher than that of 2 mg/L KIN (4.82 ± 0.47 g/L). The positive impact of GA and JA supplementation on DHA production was also found in other thraustochytrids [20,31]. It was found that 4 mg/mL of GA increased the DHA yield of *Aurantiochytrium* sp. YLH70 up to 79.1% by accelerating the rate of glucose utilization, and inducing higher production of metabolites involved in fatty acids biosynthesis, especially those related to the PKS pathway, explaining the significant enhancement in DHA biosynthesis. In addition, Wang et al. [31] demonstrated that an increment of 12.71% lipid was observed when *Schizochytrium* sp. S31 was supplemented with 20 mg/L of JA, and the combination of JA and BNAO further increased the lipid production in *Schizochytrium* sp. S31 by 20%. Different studies on microalgae have also supported this observation where the combination of different classes of phytohormones further enhanced the growth and lipid production in *Nannochloropsis* spp., *Scenedesmus* spp., *Chlorella* spp., etc. [26,27,32]. However, most of the previous studies directly combined different types of phytohormones with the optimal concentration obtained from individual treatment. Although some studies have obtained positive outcomes, the direct combination of phytohormones using the optimal concentration obtained from the individual study may not result in optimal outcomes, leading to a detrimental impact on the cell. For example, a direct combination of cytokinin and auxin with the concentration obtained from the individual experiment led to a decrease in lipid and arachidonic acid (ARA) production by 54% and 64% in *Montrella alpina* [33]. Therefore, in order to address this issue, the RSM was deployed. The RSM is a useful statistical optimization design that has successfully been used in many biological and chemical processes [34,35,36]. The RSM creates an experimental design with the minimum number of experiments and generates a model that can predict the interaction and correlation between a set of independent variables and observed results, thus providing optimized conditions that differ from the conventional approach of optimization, which only investigates one factor at a time.

### 3.2. Synergistic Effect and Optimization of KIN, JA and GA for Enhanced Growth and DHA Production of SW1

Since the individual supplementation of KIN, JA and GA (Figure 1) only exhibited a selectively significant (*p* < 0.001) impact on either enhancing the growth or the DHA production, it is interesting to evaluate whether the synergistic supplementation of these phytohormones could potentially enhance both biomass and DHA production in SW1. Thus, the synergistic impact of these phytohormones was determined using an RSM based on a central composite design (RSM-CCD). The range of concentration of all the three phytohormones was chosen based on the screening experiment as shown in Figure 1. Twenty sets of experiments at a different combination of factors were performed and the mean result of the three replicates for biomass and DHA production (experimental and predicted) are presented in Supplementary Table S1.

**Figure 2:**
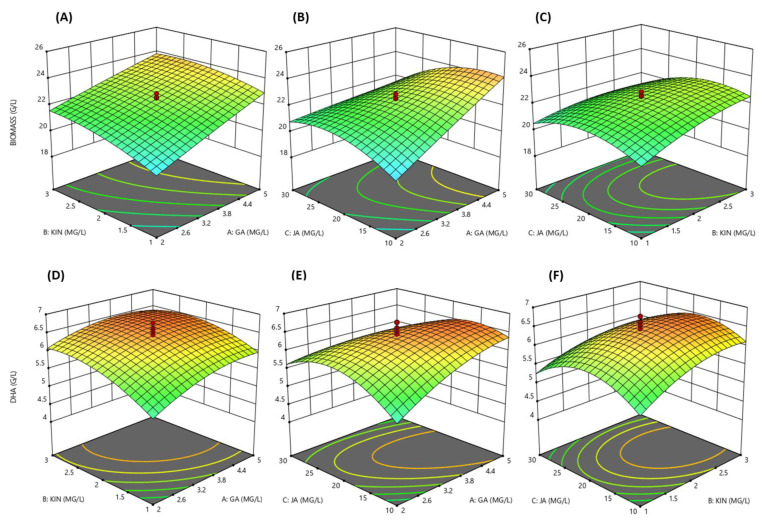
3D response surface visualization for the synergistic effect of KIN, JA and GA on biomass (**A**–**C**) and DHA (**D**–**F**) production by SW1.

These results showed that the synergistic combination of KIN, JA and GA significantly affects the growth and DHA productions of SW1 (Supplementary Table S1). The three-dimensional (3D) response surface was plotted to study the interaction of the three phytohormones on the growth and DHA production of SW1 (Figure 2A–F). This type of graphical visualization allows the relationships between the experimental levels of each factor, the response and the type of interactions between the test variables, which is necessary to establish the optimal medium components and culture conditions. Based on the 3D surface and results shown in Supplementary Table S1, the maximum biomass production of 25.20 g/L was achieved at run no. 10 with the combination of 6.02 mg/L GA, 2 mg/L KIN and 20 mg/L JA, while the maximum DHA production (6.52 g/L) was achieved at run no. 15 with the combination of 3.5 mg/L GA, 2 mg/L KIN and 20 mg/L JA, indicating that different concentrations of these phytohormones had a different impact on the biomass and DHA production of SW1. Thus, it is important to obtain the optimal concentration of these phytohormones for the maximum production of both biomass and DHA. In order to achieve these objectives, further optimization was conducted.

Five models were tested, namely mean, linear, 2FI, quadratic and cubic; among all, the quadratic model was chosen to further analyze the data as it had the highest-order polynomial with a significant sum of squares, an insignificant lack-of-fit and the highest R^2^.

Based on the ANOVA analysis (Supplementary Table S2), the experimental values obtained from the design were regressed using a quadratic polynomial equation, and the regression equation, expressed in terms of the actual factors, are shown below.

Biomass (g/L) = 5.60204 + 2.71256 × GA + 3.6008 × KIN + 0.66474 × JA − 0.025696 × GA^2^ − 0.40253 × KIN^2^ − 9.66448 × 10^−3^ × JA^2^ − 0.15000 × GA × KIN − 0.066667 × GA × JA − 0.039750 × KIN × JA(2)

DHA (g/L) = −0.85194 + 1.50295 × GA + 1.91927 × KIN + 0.32365 × JA − 0.10141 × GA^2^ − 0.28490 × KIN^2^ − 5.71439 × 10^−3^ × JA^2^ − 0.070435 × GA × KIN − 0.024087 × GA × JA − 0.011022 × KIN × JA(3)

Based on the regression analysis of the model equation for biomass and DHA production, the optimum levels of the variables were estimated. The optimum conditions proposed by the model were 3.6 mg/L GA, 2.0 mg/L KIN and 20.0 mg/L JA with the predicted optimal biomass and DHA production of 24.39 and 6.55 g/L, respectively. Validation of the predicted value was carried out and it was found that SW1 produced 24.21 g/L biomass and 6.56 g/L DHA in agreement with the predicted model, and these concentrations were 48.70% and 126.21% higher than those of the control, indicating that the RSM was successfully applied to enhance the growth and DHA production in SW1. In addition, the optimal combination GA, KIN and JA was also shown to be better than the individual supplementation of the phytohormones (Table 2).

A similar result has also reported by Renuka et al. [27], where the combination of 0.5 mg/L zeatin, 1.0 mg/L indole acetic acid and 5.0 mg/L GA enhanced the growth and lipid production of *Acutodesmus obliquus* by 49.0% and 77.20%, respectively, as compared to the control. In addition, the combination of KIN and GA exhibited a synergistic effect which resulted in a twofold increase in total lipid yield and a fourfold increment in EPA percentage in *N. oceanica* CASA CC201 [26]. In line with the result obtained in this study, the synergistic impact of SA and ETA in *C. cohnii* was shown to enhance lipid production by 20% in comparison to individual supplementation with the phytohormones [28]. In another study, Wang et al. [31] reported that the impact of phytohormone supplementation was not only shown to be dose-dependent but also time-dependent, where supplementation of 2 mg/L BNOA and 20 mg/L JA at 48 h of cultivation further increased lipid accumulation of *Schizochytrium* sp. S31 up to 16.79% compared to 0 h. Therefore, further study was conducted to elucidate the impact of the optimal concentration of KIN, JA and GA obtained in this study at different time points.

### 3.3. Influence of the Combined Addition Strategy on the Growth, Lipid and DHA Production in SW1 at Different Feeding Time Points

**Figure 3:**
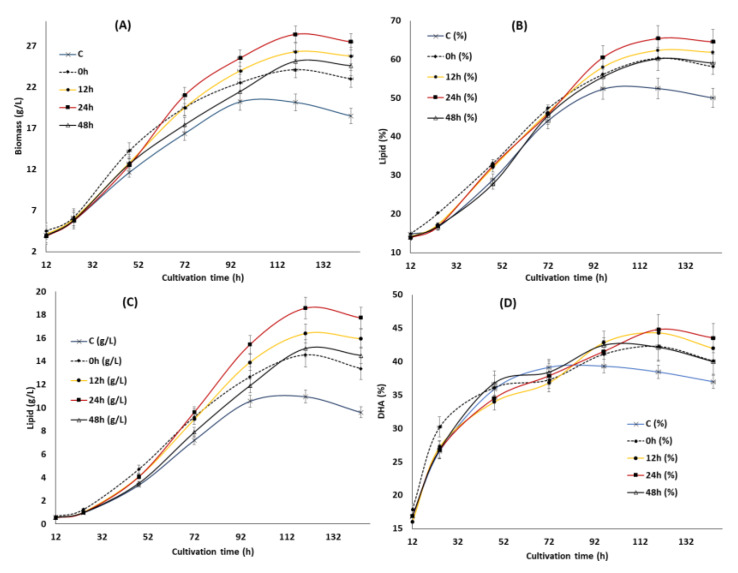

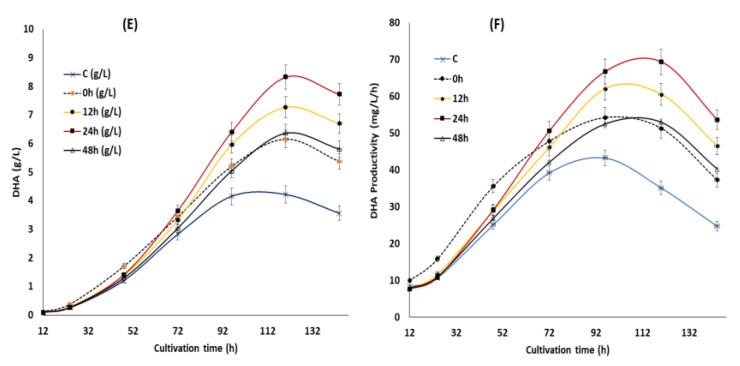
The impact of the synergistic optimal concentration of KIN, JA and GA on biomass (**A**), lipid (**B**,**C**) and DHA (**E**,**F**) at the 0, 12, 24 and 48 h feeding points in SW1.Values and error bars represent the means and the standard deviations of triplicate experiments (*n* = 3).

To further improve the inducing effect of the optimized KIN, JA and GA obtained from the RSM study on growth, lipid and DHA production, the suitable feeding time points were investigated. Four different time points, namely 0, 12, 24 and 48 h (regarded as the 0, 12, 24, and 48 h groups henceforth), were selected and the impact on the biomass, lipid and DHA production was investigated (Figure 3). These time points were selected based on its involvement in growth, lipid and DHA production as reported in the previous study [34]: 0 h—starting point of the experiment, 12 h—lag phase, 24 h—early log phase and 48 h—nitrogen exhaustion point. The result showed that supplementation of KIN, JA and GA at different time points gave a significant impact on the growth of SW1 where the maximum impact was observed at 24 h and the minimum was observed at 0 h of cultivation. Feeding of KIN, JA and GA at 24 h was shown to improve the biomass concentration by 50.23% as compared to the control as well as 15.25%, 15.20% and 7.94% in comparison to the 0, 48 and 12 h groups, respectively (Figure 3 and Table 3). Nevertheless, the reduction in biomass production observed in the group supplemented with all the phytohormones after 120 h of cultivation was probably due to the exhaustion of glucose in the cultivation media (data not shown).

Furthermore, the pattern of the lipid accumulation was also shown to be similar to the biomass as the maximum lipid content of 65.41% was achieved in the 24 h group after 120 h of cultivation (Figure 3B). Nevertheless, no significant (less than 5%) difference in lipid content (%) was observed between the 24 h and 12 h groups and the greater lipid production (g/L) observed in the 24 h group (Figure 3C) was predominantly due to the differences in growth. However, the lipid content (%) in the 24 h group was 28%, 8.5% and 9% higher than that of the control, 0 and 48 h of feeding (Figure 3B). The result obtained in the study was different from what was reported by Wang et al. [31], where the maximum impact in *Schizochytrium* sp. S31 was observed at 48 h of cultivation. This phenomenon might be rationalized by the differences in strains and phytohormones used in the study. However, the cause of lipid turnover (Figure 3B) observed between 96 h to 144 h in the control group was not obvious since glucose was still available in the cultivation media (data not shown). Lipid turnover commonly occurs in oleaginous microorganisms after the exhaustion of carbon sources where the storage lipid is used to support the viability of the cells through β-oxidations [37]. Only a slight lipid turnover was observed in the phytohormone supplemented groups, even after the exhaustion of glucose (after 120 h), indicating there was a difference in the cellular metabolism for lipid accumulation between the control and phytohormone supplemented groups in SW1.

Supplementation of the optimized KIN, JA and GA at different time points also had a significant impact on the DHA content of SW1 between the phytohormone supplemented group and the control, especially in the later stage of cultivation. Even though there was no substantial difference in the DHA content (%) of SW1 in all the phytohormone supplemented groups, the DHA production (g/L) in the 24 h group was 35.23%, 14.58% and 30.77% higher than that of the 0, 12 and 48 h, respectively. In addition, a pronounced impact was also observed in the DHA productivity of the 24 h group, reaching up to 69.42 mg/L/h at 120 h of cultivation, and 105%, 35%, 14% and 30% than that of the control, 0, 12 and the 48 h groups, respectively. Therefore, it was proposed that supplementation of the optimal concentration of KIN, JA and GA is ideal at 24 h of cultivation, to further improve the inducing effect of the phytohormones on growth, lipid and DHA production in SW1. Nevertheless, the potential mechanisms explaining the results observed in this study are still elusive. Therefore, further study was conducted to evaluate the potential mechanism of phytohormone treatment at different time points. Several studies reported that supplementation of phytohormones in microalgae was shown to reduce the stress response of the cells by enhancing the activities of the antioxidant related enzymes, such as SOD and CAT [16,17,18,19]. Since the reduction of the stress levels and enhancement of the antioxidant enzymes was shown to directly increase the level of PUFA in *Schizochytrium* and many other microalgae, it is worth evaluating its involvement in enhancing the growth, lipid and DHA production in SW1.

### 3.4. Influence of the Synergistic Optimal Concentration of KIN, JA and GA at Different Time Points on the Antioxidant Defense Systems of SW1

**Figure 4:**
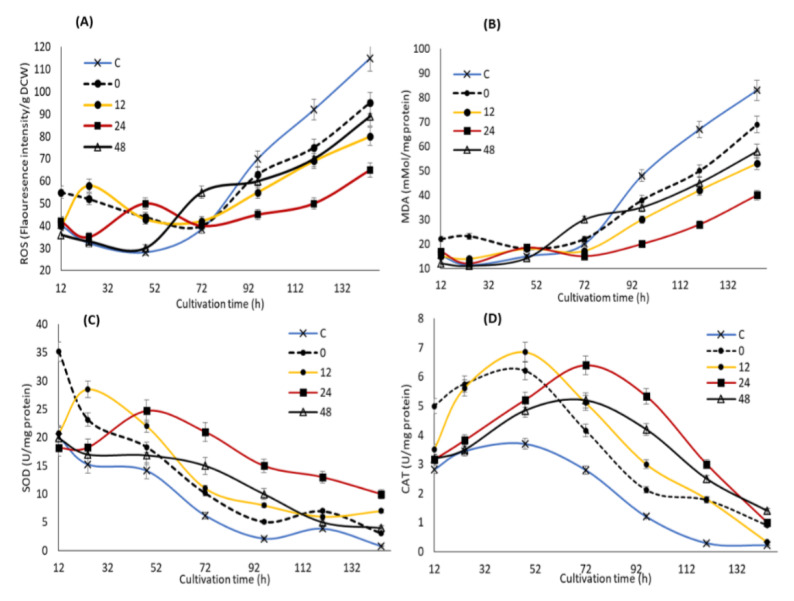
The impact of the synergistic optimal concentration of KIN, JA and GA on ROS (**A**), MDA (**B**), SOD (**C**) and CAT (**D**) at the 0, 12, 24 and 48 h feeding points in SW1. Values and error bars represent the means and the standard deviations of triplicate experiments (*n* = 3).

In order to elucidate the potential impact of KIN, JA and GA supplementation at different time points on the stress markers and oxidative defense systems of SW1, the concentrations of ROS and MDA, as well as the activities of SOD and CAT were determined. Cellular ROS comprises primarily superoxide anion (O^2−^), hydrogen peroxide (H_2_O_2_), hydroxyl radical (·OH), lipid hydroperoxides (LOO) and peroxyl radicals (LOO·) [38]. Our results showed that feeding of the phytohormones at different time points exhibited a different impact on the stress marker and antioxidant enzymes of SW1 (Figure 4). During the early to mid-stage of the cultivation (12–72 h), supplementation of the phytohormones was shown to immediately increase the ROS at a moderate level (from 30 to 60 fluorescence intensity/g CDW), approximately 0.5–1.5-fold higher than that of the control group. According to Piotrowska et al., [39] as well as Sivaramakrishnan and Incharoensakdi [40], addition of exogenous phytohormones induces an increase in the cellular respiration of microalgae, which in turn led to the increased production of ROS. This explained the higher cellular growth in the phytohormone supplemented group of SW1 in comparison to the control, as the importance of higher cellular respiration in the early stage of cultivation in promoting cellular growth and lipid production in thraustochytrids has been established [41,42]. In addition, previous studies have also reported that a moderate level of ROS was important to promote cell differentiation and proliferation in *Chlorella* spp. by acting as a signaling molecule that improves the cell growth and physiological responses [40]. Nevertheless, the ROS levels should be maintained at a moderate level as elevated ROS will be detrimental to the cells. In this study, the ROS levels in the groups supplemented after 0, 12 and 24 h were immediately lowered after the induction as the cells were still able to produce ample antioxidants to resist the oxidative stress. On the other hand, a different trend of the ROS level was observed when the phytohormones were supplemented at 48 h of cultivation where the deactivation of ROS was shown to be minimal in comparison to the other groups.

This phenomenon could be explained by observing the impact of the phytohormones treatment on the level of the antioxidative enzymes (Figure 4). The results showed that the moderate increment of ROS induced by the phytohormones treatment significantly enhance the activity of SOD and CAT enzymes, particularly at 24 h, which was ideal to maintain the activity of both enzymes at the elevated level throughout the cultivation. A similar result has also been reported by Sivaramakrishnan and Incharoensakdi [40], in that addition of 1 mM IAA and ABA resulted in ROS generation, which in turn improved the antioxidant enzymes (SOD and CAT) levels. Although feeding at the early stage of cultivation (0 and 12 h) triggered higher activity of the oxidative defense enzymes, the activities quickly weakens after 72 h of cultivation. On the other hand, only a minimal impact of both SOD and CAT was observed when feeding at 48 h of cultivation, explaining the minimal reduction of the ROS level after the induction. Nevertheless, the activity of SOD and CAT of the 48 h group remain significantly higher than the control throughout the cultivation. During the mid to late stage of the cultivation (72–144 h), a significant increment of the ROS level reaching up 120 fluorescence intensity/mg DCW was observed in the control group, as the cell was unable to resist the oxidative stress due to the diminishing levels of its SOD and CAT enzymes activities. A similar trend was also observed in all the phytohormones supplemented groups. Nevertheless, the ROS level of SW1 in all the phytohormones supplemented groups was significantly lower than that of the control group as they pose a higher antioxidant activity. In accordance with this study, Beck et al. [43] has proposed that ABA could participate in the ROS signaling pathway by regulating ROS production and clearance by modulating the oxidative stress regulatory mechanisms.

Severe oxidative stress due to elevated levels of ROS and the significant decline of the antioxidant enzymes may lead to lipid peroxidation as indicated by the increase in MDA levels, a major indicator of lipid peroxidation. Our results showed that the MDA concentration was maintained at a low level at the early stage of fermentation but significantly increased after 72 h of cultivation, and coincided with the elevation of ROS in all groups except at 24 h where the MDA level was maintained at lower than 45 mMol/mg protein throughout the cultivation. In addition, the control group reached a maximum MDA level of 90 Mmol/mg protein, which was 24%, 50%, 58% and 100% higher than that of the 0, 48, 12 and 24 h groups, respectively. Lipid peroxidation has been established to cause oxidative degradation of lipids especially the cell membranes as well as the polyunsaturated fatty acids (PUFAs), resulting in cellular damage and reduction in the overall PUFA content [44]. These conditions might explain the early onset of lipid and DHA turnover observed in the control group, as well as higher growth, lipid and DHA production in the phytohormones-supplemented group, especially the 24 h. The result of this study also suggested that supplementation of the optimal concentration of KIN, JA and GA at 24 h of cultivation was ideal in mitigating oxidative injury through enhancing the activity of antioxidant enzymes. Similarly, some evidence indicated that phytohormones can regulate the oxidative stress response in microalgae where the addition of ABA can greatly reduce the oxidative stress in *Chlamydomonas reinhardtii* through enhancing the CAT and APX activities [45]. Consistently, in *Chlorella vulgaris*, the greatest enhancements in antioxidant activities can be attributed to the addition of indole-3-acetic acid [39], in line with the result of this study.

### 3.5. Influence of the Synergistic Optimal Concentration of KIN, JA and GA on the Key Metabolic Enzymes Involved in Growth and Fatty Acid Biosynthesis in SW1 at Different Feeding Time Points

**Figure 5:**
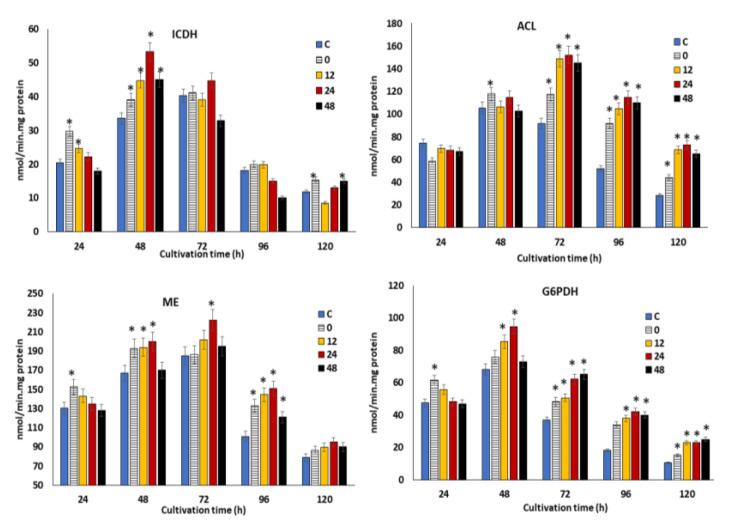
The impact of the synergistic optimal concentration of KIN, JA and GA on ICDH, ACL, ME and G6PDH at the 0, 12, 24 and 48 h feeding points in SW1.Values and error bars represent the means and the standard deviations of triplicate experiments (*n* = 3). (*) represents the statistical significance with respect to control values at *p* < 0.05.

In the previous section, supplementation of the optimal concentration of KIN, JA and GA at different time points was shown to modulate the oxidative stress, which could directly promote the growth, lipid and DHA production in SW1. However, the potential impact of the phytohormones treatment at different time points on the key metabolic enzymes involved in the growth and fatty acid biosynthesis in SW1 remains vague. Therefore, the profiles of the specific activities of four key enzymes, namely glucose-6-phosphate dehydrogenase (G6PDH), malic enzyme (ME) NAD: isocitrate dehydrogenase (ICDH) and ATP: citrate lyase, were determined (Figure 5). The ICDH activity was determined due to its importance and implication in the initiation of lipogenesis. Upon cessation of growth and initiation of lipogenesis, the flux of carbon is diverted to lipid synthesis through the diminishing activities of ICDH [46]. Therefore, the changes in the specific activities of this enzyme could be correlated to growth and lipogenesis. Figure 5 shows the profiles of specific activities of the enzymes throughout the cultivation period. Maximum impact was observed in the 24 h group, achieving 55 nmol/mg protein at 48 h of cultivation, which is 38%, 20% and 22% higher than that of the 0, 12 and 48 h groups, respectively. This result explained the higher growth achieved in the 24 h phytohormone supplemented group. This result agrees with the finding by Geng at al [47] and Tang et al. [46], in that the higher growth observed in the engineered strain of *Schizochytrium* and *Mucor circinelloides* WJ11 coincide with higher ICDH activity. The activity of ICDH, however, rapidly decreased during the lipid accumulation phase in the control and all the phytohormone treatment groups.

The decrement in the ICDH activity subsequently results in the accumulation of citrate within the mitochondria, which was then transported into the cytosol and cleaved by ACL to generate oxaloacetate and acetyl-CoA. The acetyl-CoA was then used as a precursor for fatty acid biosynthesis. The specific activity of ACL in all the phytohormone supplemented groups reached the maximum level at 72 h and remained elevated throughout the cultivation. It was also found that supplementation of the phytohormones at 12, 24 and 48 h resulted in maximum ACL activity, which was 20%–60% higher than the 0 h and control group, implying a higher production of acetyl-CoA, and which resulted in a higher lipid content (as shown in Figure 3B).

Beside acetyl-CoA, the provision of reducing power in the form of NADPH is another critical process for fatty acid biosynthesis, which is usually attributed to two pathways: the pentose phosphate pathway (PPP) and the malic acid/pyruvate shuffle pathway [16]. Precisely, the G6PDH, 6PGDH and ME are the key enzymes of the two respective pathways, which are directly related to the supply of NADPH. As illustrated in Figure 5, SW1 exhibited a 40%–60% higher ME activity in all the phytohormone supplemented groups, where the maximum activity of 230 nmol/min/mg protein was achieved in the 24 h group at 72 h of cultivation, 15%–43% higher than the control and other feeding time points. A similar profile was also observed in G6PDH activities, as supplementation with phytohormones at 24 h posed a 18%–60% higher activity between 48 h to 96 h in comparison to the control and other phytohormone treatment time points. From these results, it was clearly demonstrated that supplementation with phytohormones at 24 h resulted in a higher provision of NADPH than that of the control and other time points, explaining its higher lipid and overall DHA biosynthesis capacity. In line with this study, treatment with different phytohormones, such as zeatin, IAA, GA and ABA, also resulted in increased expression of genes involved in fatty acid biosynthesis in *Chlorella* spp. [40]. In addition, Sun et al. [16] also reported that the combined treatment of fulvic acid and EDTA resulted in higher ME and lower phosphoenolpyruvate carboxylase activities in comparison to the control, explaining the significantly greater lipid yield in the phytohormone treatment group of *Schizochytrium* spp.

## 4. Conclusions

In this study, three phytohormones (KIN, JA and GA) with a significant impact on the growth and DHA production of SW1 were identified by screening eight phytohormones from six different classes. RSM software was employed to obtain the synergistic optimal concentration of 3.6 mg/L GA, 2.0 mg/L KIN and 20.0 mg/L JA, which resulted in a 48% and 126% increment in growth and DHA production in comparison to the control, respectively. The synergistic effect of these phytohormones was also shown to be time-dependent, where feeding of the phytohormones at 24 h of cultivation led to 15%, 26% and 35% further increments in the biomass, lipid and DHA production in comparison to that of 0 h, respectively. Feeding at 24 h after cultivation was shown to exhibit the maximum impact in reducing the levels of ROS and MDA, as well as augmenting the antioxidants (SOD and CAT) and key metabolic enzymes involved in lipogenesis (ME, G6PDH, ACL and ICDH), demonstrating the potential application of phytohormones for improving the growth, lipid and DHA production in *Aurantiochytrium* spp.

## Figures and Tables

**Table 1:** List of phytohormones, classes and concentrations used in this study.

Phytohormones		Concentration (mg/L)						
Name	Classes							
Naphthoxyacetic acid (BNOA)	Auxin	0.5	1	2	5	10	20	40
Gibberellic acid (GA)	Gibberellin	0.5	1	2	3	4	5	8
Kinetin (KIN)	Cytokinin	0.2	0.5	1	2	3	4	5
Salicylic acid (SA)	Signal Transducer	0.5	1	2	5	10	20	40
Jasmonic acid (JA)		1	2	5	10	20	40	50
Abscisic acid (ABA)		0.5	1	2	5	10	20	40
Melatonin	Melatonin	0.2	0.5	1	2	3	4	5
Ethanolamine (ETA)	Amines	0.5	1	5	10	20	50	200

**Table 2:** Comparison of biomass, DHA production and percentage increment, with the control set as the reference, by SW1 after 96 h of cultivation supplemented with the synergistic optimal concentration of KIN, JA and GA. Values represent the means and the standard deviations of triplicate experiments (*n* = 3).

Treatments	Concentration (mg/L)	Biomass (g/L)	DHA (g/L)	Percentage Increment (%)	
				Biomass	DHA
Control	-	16.27 ± 1.55	2.90 ± 0.24	-	-
KIN	2	20.81 ± 1.92	4.82 ± 0.47	27.90 ± 2.63	66.20 ± 4.60
JA	20	18.93 ± 1.74	5.10 ± 0.44	16.34 ± 1.54	75.86 ± 4.78
GA	4	19.55 ± 1.80	5.34 ± 0.43	20.16 ± 1.90	84.14 ± 5.10
KIN + JA + GA	2 + 20 + 3.61	24.21 ± 2.23	6.56 ± 0.55	48.70 ± 4.60	126.21 ± 8.33

**Table 3:** Comparison of biomass, lipid, DHA content, yield and biosynthetic capacity of SW1 after 120 h of cultivation supplemented with the synergistic optimal concentration of KIN, JA and GA at different time points. Values and standard deviations represent the mean data of triplicate experiments (*n* = 3).

Parameters	Units	Control	0 h	12 h	24 h	48 h
Biomass	g/L	19.27 ± 1.55	25.12 ± 1.34	26.82 ± 1.50	28.95 ± 1.93	25.13 ± 1.42
Lipid	g/L	10.57 ± 0.59	14.62 ± 0.82	16.80 ± 0.94	18.52 ± 1.04	15.11 ± 0.85
DHA	g/L	4.06 ± 0.23	6.16 ± 0.44	7.27 ± 0.41	8.33 ± 0.50	6.37 ± 0.36
DHA productivities	mg/L/h	33.86 ± 1.90	51.30 ± 2.87	60.58 ± 3.39	69.42 ± 3.90	53.06 ± 2.97
DHA biosynthetic capacity	(g/L DHA/g/L Biomass)	0.20 ± 0.01	0.25 ± 0.01	0.27 ± 0.01	0.29 ± 0.01	0.25 ± 0.01

## Supplementary Materials

The following are available online at https://www.mdpi.com/2218-273X/10/5/755/s1, Table S1: Synergistic effect of KIN, JA and GA on the biomass and DHA production using RSM, Table S2: ANOVA analysis for biomass and DHA production by RSM.
