# Supplementary material for: Different Classes of Phytohormones Act Synergistically to Enhance the Growth, Lipid and DHA Biosynthetic Capacity of Aurantiochytrium sp. SW1
Source: Biomolecules. 2020 May 13;10(5):755. doi: 10.3390/biom10050755 (PMC7277723; doi:10.3390/biom10050755)
Supplement: Supplementary file 1 [file biomolecules-10-00755-s001.pdf]

Supplementary File

Different classes of phytohormones act synergistically to enhance growth, lipid and DHA biosynthetic capacity of *Aurantiochytrium* sp. SW1

Yusuf Nazir[1], Hafiy Halim[1], Pranesha Prabankaran[1], Xiaojie Ren[1], Tahira Naz[1], Hassan Mohamed[1,2] Shaista Nosheen[1], Kiren Mustafa[1], Aidil Abdul Hamid[3*] & Yuanda Song[1*]

[1] Colin Ratledge Center for Microbial Lipids, School of Agriculture Engineering and Food Science, Shandong University of Technology, Zibo 255049, China.

[2] Department of Botany and Microbiology, Faculty of Science, Al-Azhar University, Assiut, 71524, Egypt.

[3]School of Biosciences and Biotechnology, Faculty of Science and Technology, Universiti Kebangsaan Malaysia

* Correspondence authors: Yuanda Song & Aidil Abdul Hamid

Email: ysong@sdut.edu.cn (Yuanda Song), aidilmikrob@gmail.com (Aidil Abdul Hamid)

Supplementary Table 1: Synergistic effect of KIN, JA and GA on the biomass and DHA production using RSM.

Std. Order	Variables			Results			
				Experimental Values		Predicted Values	
	GA (mg/L)	KIN (mg/L)	JA (mg/L)	Biomass (g/L)	DHA (g/L)	Biomass (g/L)	DHA (g/L)
1	2	1	10	18.21	4.35	17.77	4.31
2	5	1	10	22.81	5.50	22.92	5.76
3	2	3	10	20.58	4.97	20.36	5.37
4	5	3	10	24.18	6.28	24.61	6.39
5	2	1	30	20.46	4.88	19.87	5.03
6	5	1	30	20.96	5.17	21.02	5.03
7	2	3	30	21.14	5.64	20.87	5.65
8	5	3	30	20.84	4.92	21.12	5.23
9	0.977	2	20	19.24	5.55	20.06	5.36
10	6.02	2	20	25.20	6.41	24.60	6.22
11	3.5	0.32	20	19.80	5.12	20.23	5.10
12	3.5	3.68	20	22.69	6.52	22.48	6.15
13	3.5	2	3.18	20.35	5.32	20.34	5.10
14	3.5	2	36.82	18.95	4.70	19.18	4.63
15	3.5	2	20	22.61	6.52	22.50	6.43
16	3.5	2	20	21.90	5.95	22.50	6.43
17	3.5	2	20	22.45	6.21	22.50	6.43
18	3.5	2	20	22.55	6.53	22.50	6.43
19	3.5	2	20	22.60	6.45	22.50	6.43
20	3.5	2	20	22.90	6.57	22.50	6.43

Supplementary Table 2; The ANOVA analysis for biomass and DHA production by RSM.

	Biomass					DHA				
**Sources**	**Sum of Squares**	**DF**	**Mean Square**	**F Value**	**Prob > F**	**Sum of Squares**	**DF**	**Mean Square**	**F Value**	**Prob > F**
Model	57.20	9	6.36	22.75	< 0.0001	9.43	9	1.05	9.17	0.0009
A	24.85	1	24.85	88.96	< 0.0001	0.89	1	0.89	7.81	0.0190
B	6.14	1	6.14	21.99	0.0009	1.34	1	1.34	11.68	0.0066
C	1.64	1	1.64	5.87	0.0358	0.17	1	0.17	1.52	0.2460
A^2	0.048	1	0.048	0.17	0.6867	0.75	1	0.75	6.56	0.0283
B^2	2.34	1	2.34	8.36	0.0161	1.17	1	1.17	10.23	0.0095
C^2	13.46	1	13.46	48.18	< 0.0001	4.71	1	4.71	41.17	< 0.0001
AB	0.41	1	0.41	1.45	0.2563	0.089	1	0.089	0.78	0.3975
AC	8.00	1	8.00	28.63	0.0003	1.04	1	1.04	9.14	0.0128
BC	1.26	1	1.26	4.52	0.0593	0.097	1	0.097	0.85	0.3782
Residual	2.79	10	0.28			1.14	10	0.11		
Lack of Fit	2.25	5	0.45	4.11	0.0736	0.70	5	0.14	1.60	0.3091
Pure Error	0.55	5	0.11			0.44	5	0.088		
Cor Total	59.99	19				10.57	19			
